# The repercussions of watching scenes of the escalating conflict in Gaza strip on the mental health of adolescents in a neighboring country

**DOI:** 10.1186/s12889-025-22550-5

**Published:** 2025-04-30

**Authors:** Mira M. Abu-Elenin, Mohamed M. Radwan, Mohamed M. Rabie, Mohamed M. Eldabaa, Mohammed M. Abd El Wahab, Yousef M. Shatat, Mohamed M. Taalap, Mohamed M. El Sabry, Reham M. Mounir

**Affiliations:** 1 Present Address: Public Health and Community Medicine Department, Faculty of Medicine, Tanta University, Tanta City, Egypt; 2 Graduate medical researcher, Faculty of Medicine, Tanta University, Tanta City, Egypt

**Keywords:** Adolescents, Conflict, Gaza, Media, Mental health, War

## Abstract

**Background:**

Over the past decade, prompt technological innovation has accelerated the news dissemination of armed conflict and wars through various media channels, yielding mass fear, anxiety, and depression. Adolescents are more susceptible to experiencing mental distress as a result of watching such uncensored scenes.

**Aim:**

This study aimed to assess the psychological impact of exposure to conflict scenes in the Gaza Strip on the mental health of school adolescents.

**Methods:**

A cross-sectional study involved 519 adolescents aged 11–18 years, recruited through clustered sampling technique from private and public middle and high schools in Gharbia governorate, Egypt. An anonymous self-administered questionnaire was deployed and included the Arabic version of the depression, anxiety, and stress scale (DASS21).

**Results:**

Around 30% of the studied adolescents were diagnosed as stressed, 61.5% were depressed, and 57% were anxious. These adverse mental outcomes were more prevalent among females and adolescents in high schools. Additionally, participants who were regularly exposed to conflict scenes for an average of 5–7 days per week, with a viewing duration exceeding 3 h per day, were more likely to experience these mental issues.

**Conclusions:**

The study unveiled a high prevalence rate of stress, depression, and anxiety among adolescents. This observation was positively associated with the frequency and intensity of media exposure to the conflict scenes in the area. Supportive initiatives and controlling exposure to media for adolescents in conflict zones have unequivocal value in ameliorating mental disorders.

## Background

The last year, 2023, has witnessed incidents of armed conflicts across the globe; the most recent is the escalated conflict in the Gaza Strip, which presents a set of unique challenges differing from the past era confrontations in both fierceness and drastic impact on inhabitants due to the holistic damage of infrastructure and brutal assaults [1].

International conflicts refer to disputes or disagreements between two or more sovereign states, often involving differences in interests, values, or territorial claims. These conflicts can manifest through diplomatic tensions, economic sanctions, military confrontations, or other forms of hostilities on the global stage. Conflicts and wars potentially reflect on adolescents, who transit from childhood to adulthood with marked developmental, social, mental, and behavioural changes during this phase of life [2]. The World Health Organization (WHO) defines mental health as wellbeing, self-awareness, stress management, practical work, and community contribution. This definition highlights the interconnected nature of mental health within the broader context of human wellbeing and the surrounding environment [3].

Despite the global initiatives and peaceful acts for a world without violence, wars and conflicts persist [4, 5]. The adverse impact of such conflicts on mental health was starkly demonstrated in various zones, including Afghanistan, the Balkans, Cambodia, Chechnya, Iraq, Lebanon, Rwanda, Sri Lanka, Somalia, and Ukraine. The findings indicated that the duration of the conflict correlates with the severity of long-term psychological effects on the human psyche, even after the immediate conflict has subsided [4–13].

Children who witness conflicts in different parts of the world are profoundly at a high risk of developing various mental health problems, such as anxiety, depression, and Posttraumatic Stress Disorder (PTSD) [14]. On the other hand, exposure to international conflicts may also lead to desensitization and a lack of empathy towards suffering people. On the other hand, some children may feel overwhelmed and powerless in the face of such widespread violence and injustice, leading to a sense of apathy and resignation [15].

Doomscrolling describes the surge in adverse effects associated with repeated exposure to pandemic-related stories. The magnitude and duration of this effect, however, remains unclear. It is also unclear if the source of the material is associated with the magnitude of the effect. Social media may differentially impact psychopathology compared to traditional media, given differences in personal connection to the authors and possible differences in overall message valence [16].

Parents, educators, and mental health professionals must be aware of the detrimental burden of exposing children to scenes of international conflict and imperative violations. Furthermore, providing age-appropriate explanations, guidance and open discussions about the realities of war and conflict could mitigate the adverse effects on children’s psychological health. Additionally, promoting peace education and teaching children about empathy, compassion, and conflict resolution can help counteract the negative impact of doom scrolling [17].

On the international level, there is evidence of strategies to prevent or minimize children’s response to trauma in conflict zones; these are more likely to be successful through operating agencies in the area in collaboration with schools, which are the primary source of stability and safety for the children. International organizations, including the United Nations (under whose auspices all schools in the refugee camps operate) and the United Nations Children’s Fund (UNICEF), have a significant role to play in providing as much as is humanly possible [18].

The ongoing Gaza conflict, deliberate targeting, and besieging of refugee camps and hospitals have emerged as alarming tactics, profoundly affecting the Gaza population, especially vulnerable groups including women, children, and elders [19].

Moreover, the precarious situation in the Gaza Strip is due to the imperative bombing of shelters, health facilities, water sources, and power systems, which presents a peculiar challenging incident shaped by the accumulative impact of these attacks with a prolonged siege spanning over 17 years [20]. Most of Gaza’s population was cut off from food, water, and medications, where children constitute about half of its people. Meanwhile, the mortality rate among children skyrocketed either due to direct lethal trauma or disease outbreaks and a shortage of healthcare capacity [21]. The constant exposure to confrontation and restrictions has evolved into a profound, anxious environment for the surrounding populations. As an immediate neighbour, Egypt might experience potential risks and similar challenges, where the Egyptian Rafah is 5 km from the Gaza Strip, and suffers significant pressure from multiple directions. These tenses are subsequently reflected on the Egyptian population [22].

It is evidenced that children and adolescents are more likely to experience overwhelmed levels of stress, anxiety, and trauma due to exposure to wars and armed conflicts through different media channels rather than other age groups [23]. Most of the displayed scenes are ferocious and uncensored, and their watching may be unadjusted or unmonitored by adults. Additionally, aadolescents are venturesome, risk-takers, sensitive, and emotion-seeking, which can result in higher susceptibility to mental distress in armed conflict regions, where media exposure to such materials is inevitable [24, 25]. One of the elemental features of media exposure to terrorism and violence is the aggregation of several exposures to atrocious and disgraceful scenes rather than an acute single exposure [26], resulting in increased self-risk sensation and disrupting their routine daily activities [27]. Moreover, the literature elaborated that exposure to armed conflict and terroristic acts might result in unfavourable consequences such as social outrage aggression, sleep issues, isolation, discrimination, and radicalization of ideological beliefs [28].

The impact of this experience can contribute to the complexities of adolescent development. Therefore, this study aimed to highlight the adverse effects of watching Gaza conflict-related scenes through different media channels on the mental health of adolescents and potential determinants that could moderate these negative consequences.

## Subjects and methods

### Study design and settings

This observational cross-sectional study was held from November 2023 to March 2024 in Tanta City, Gharbia Governorate, Egypt. The target population of this study was school adolescents recruited using a cluster sampling technique.

The study was conducted at four randomly selected middle and high schools in Tanta city: 3 governmental schools and one private school: Badr Al-Sumaiti Hhigh School, which has six classes for each grade from grade 10 to 12, El- -Nasr Oofficial language School, which has six classes for each grade in middle and high grades of both genders, Kassem Amin High School for Girls which consists of 7 courses for each grade. Al-Salam Private Language School is a mixed-gender school that includes middle and high grades and has five classes for each grade.

In each chosen school, two classes representing grades from the 7th to the 12th grade were randomly selected; all the students in the chosen classes were enrolled in the study.

Inclusion criteria included adolescents aged between 11 and 18 years who were officially registered in the selected schools. Exclusion criteria precluded students who had any psychological health problems, as the school social specialist reported before the study was conducted, and students who were absent during determined days for data gathering.

The sample size was calculated using the CDC Epi-info program, where the margin of error was 0.05, the power of the study was 80%, the Confidence interval was 95%, and assuming that the prevalence of the outcome variable is 50% since there are no similar studies conducted yet. The computed sample was > 384, and the study expanded to enroll 519 students to ensure the representation of the adolescents in both middle and high grades.

### Study procedures and measuring tools

The investigators were allocated to the chosen schools on the assigned pre-determined days according to the schools’ managers’ guidance and the classes’ schedules. In each involved class, the investigators thoroughly explained the nature of the study and its objectives to the students. Then, the students were kindly requested to participate in the research and fulfill an anonymous self-administered questionnaire to collect the required data after being assured confidentiality and privacy.

The researchers developed the questionnaire after reviewing related literature and considering collecting the relevant data that respond promptly to the study question and help to investigate the outcome variables. The questionnaire was examined for its validity and reliability after being checked by four professional experts in community medicine, school health, and mental health; their feedback and modifications were implemented accordingly. The internal consistency coefficient (Cronbach-alpha = 0.86) and test-retest reliability were good at 78%, utilizing the SPSS program version 26.

The questionnaire consisted of three sections; the first included eight questions about sociodemographic characteristics, and the second was composed of five questions identifying the media channels, the frequency, and the duration of viewing the conflict scenes in the Gaza Strip. The last section was the Arabic version of the Depression Anxiety Stress Scale-21(DASS-21) [29], which has been extensively validated across various cultures, settings, and populations worldwide; its internal consistency coefficient ranged between 0.73 ~ and 0.91 [30, 31].

The investigators included a statement at the beginning of the questionnaire, emphasizing on participants to think deeply about each item of DASS 21 in consideration to the witnessed conflict scenes before providing their responses.

### Scoring system of DASS-21

The Depression, Anxiety, and Stress Scale—21 Items (DASS-21) is a set of three self-report scales designed to measure the negative emotional states of depression, anxiety, and stress. Each of the three DASS-21 scales contains seven items; each question has responses ranging from “0 = didn’t apply to me at all”, “1 = Applied to me to some degree, or some of the time”, “2 = Applied to me to a considerable degree, or a good part of the time”, “3 = Applied to me very much, or most of the time”.

DASS_ Anxiety items were 2, 4, 7,9,15, 19 and 20; DASS_ Depression items included 3, 5,10, 13,16, 17 and 21. At the same time, DASS_ Stress items were 1, 6, 8,11,12, 14 and 18.

The depression scale is multifactorial, assessing dysphoria, hopelessness, devaluation of life, self-deprecation, lack of interest or involvement, anhedonia, and inertia. Likely, the anxiety scale includes autonomic arousal, skeletal muscle effects, situational anxiety, and subjective experience of anxious effects. The stress scale is sensitive to levels of chronic, nonspecific arousal. Which assesses difficulty relaxing, nervous arousal, and being easily upset, agitated, irritable, overreactive, and impatient. Scores for depression, anxiety, and stress were calculated by summing up the scores for the relevant items for each participant. Scores were multiplied by 2 to calculate the final score.

The cut-off scores for conventional severity labels (regular, moderate, and severe) are as follows:

- Depression: 0–9 regular / 10–13 mild / 14–20 moderate / 21–27 severe/ +27 extremely severe.
- Anxiety: 0–7 normal / 8–9 mild / 10–14 moderate / 15–19 severe/ +20 extremely severe.
- Stress:0–14 normal /15–25 mild to moderate / 26–33 severe.

### Statistical analysis of data and data presentation

Data coding and entry were performed using a Microsoft Office Excel sheet and then exported to the Statistical Package of Social Sciences SPSS v.26, where data was cleaned, organized, tabulated, and analysed. Quantitative variables were presented as mean and standard deviation, while qualitative ones were presented as numbers and percentages. Chi *X*^*2*^ and Exact tests were the significance tests, and the *p*-value level was set at 0.05.

## Results

This study involved 519 adolescents collected from middle and high schools (36% and 64%, respectively). Their mean age was 15.75 ± 1.44 years old, ranging between 11 and 18 years old. Males were (50.3%) and females were (49.7%). Adolescents recruited from rural and urban backgrounds represented 43.7% and 56.3%, respectively. Most of their parents’ education levels ranged from middle to high (38.5% and 53.9%, respectively), and the remaining were illiterate (7.5%), as demonstrated in Table 1.

**Table 1:** Sociodemographic characteristics of the studied school adolescents (*n* = 519)

Variables	No.	%
**Gender**		
Males	261	50.3
Females	258	49.7
***Residence***		
Urban	293	56.3
Rural	226	43.7
***Education*** **Grade**		
Middle (7,8,9th)	187	36.0
High (10,11,12th)	332	64.0
***Parents’ Education Level***		
High	280	53.9
Middle	200	38.5
Illiterate	39	7.5
***Father occupation***		
Not working	61	11.8
Employee	458	88.3
***Mother Occupation***		
Not working	308	59.3
Employee	211	40.7

Most of adolescents reported watching scenes of the Gaza conflict and the aggressive attacks through various media channels, television news and talk-show programs (95.8%), while only 4.5% didn’t view any conflict scenes. About one-third of participants (35.8%) were watching these scenes at a rate of 5–7 days per week; (27%) for 3–4 days per week, and (26.6%) for 1–2 days per week, while only (10.7%) one day per week.

**Fig. 1:**
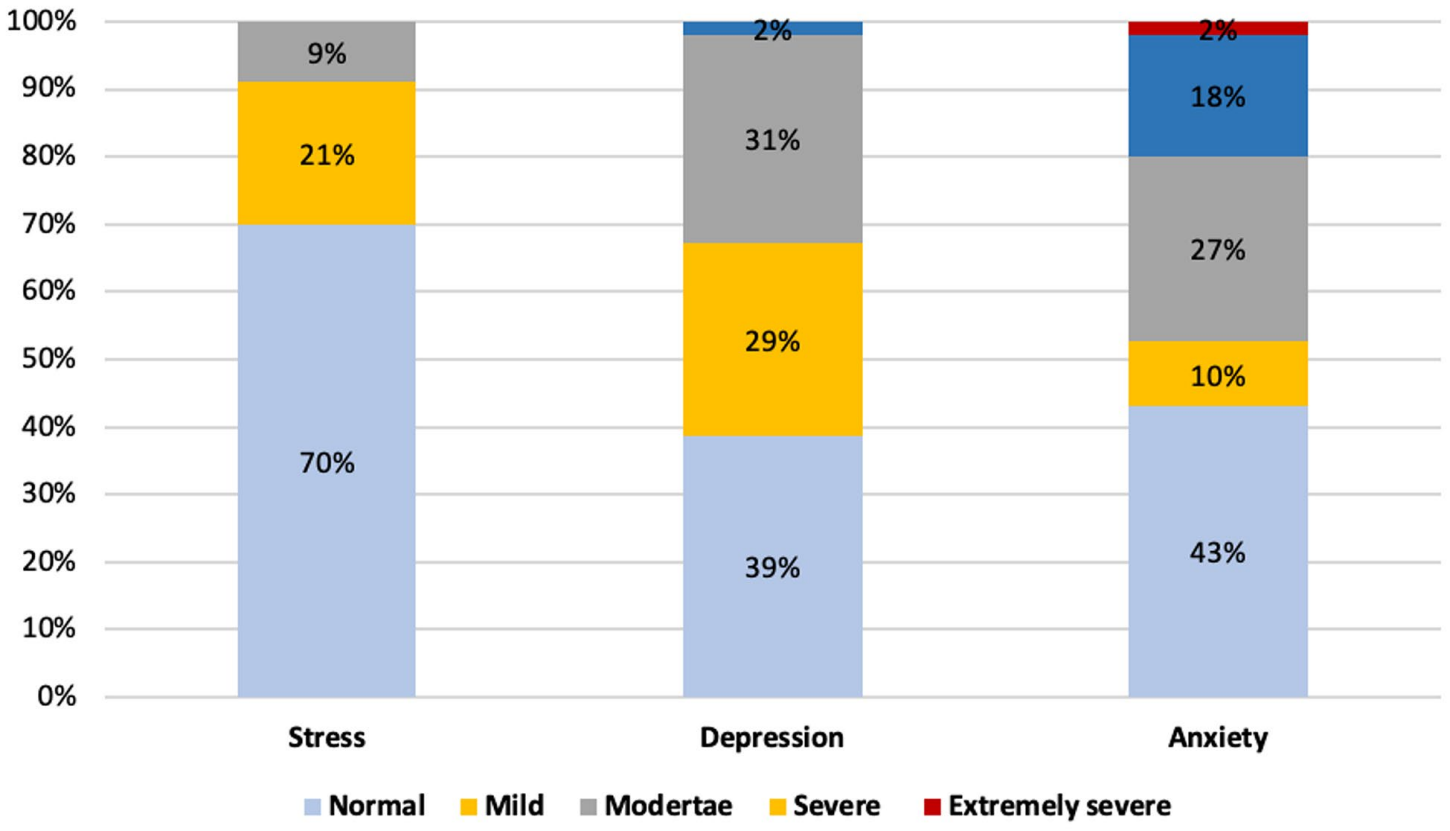
Frequency distribution of stress, depression and anxiety levels among studied school adolescents (*n* = 519)

Our findings revealed that 155 participants (29.9%) were stressed, 319 participants (61.5%) were depressed, and 296 participants (57%) were anxious, with variant degrees ranging from mild to severe level, as illustrated in Figure (1).

**Fig. 2:**
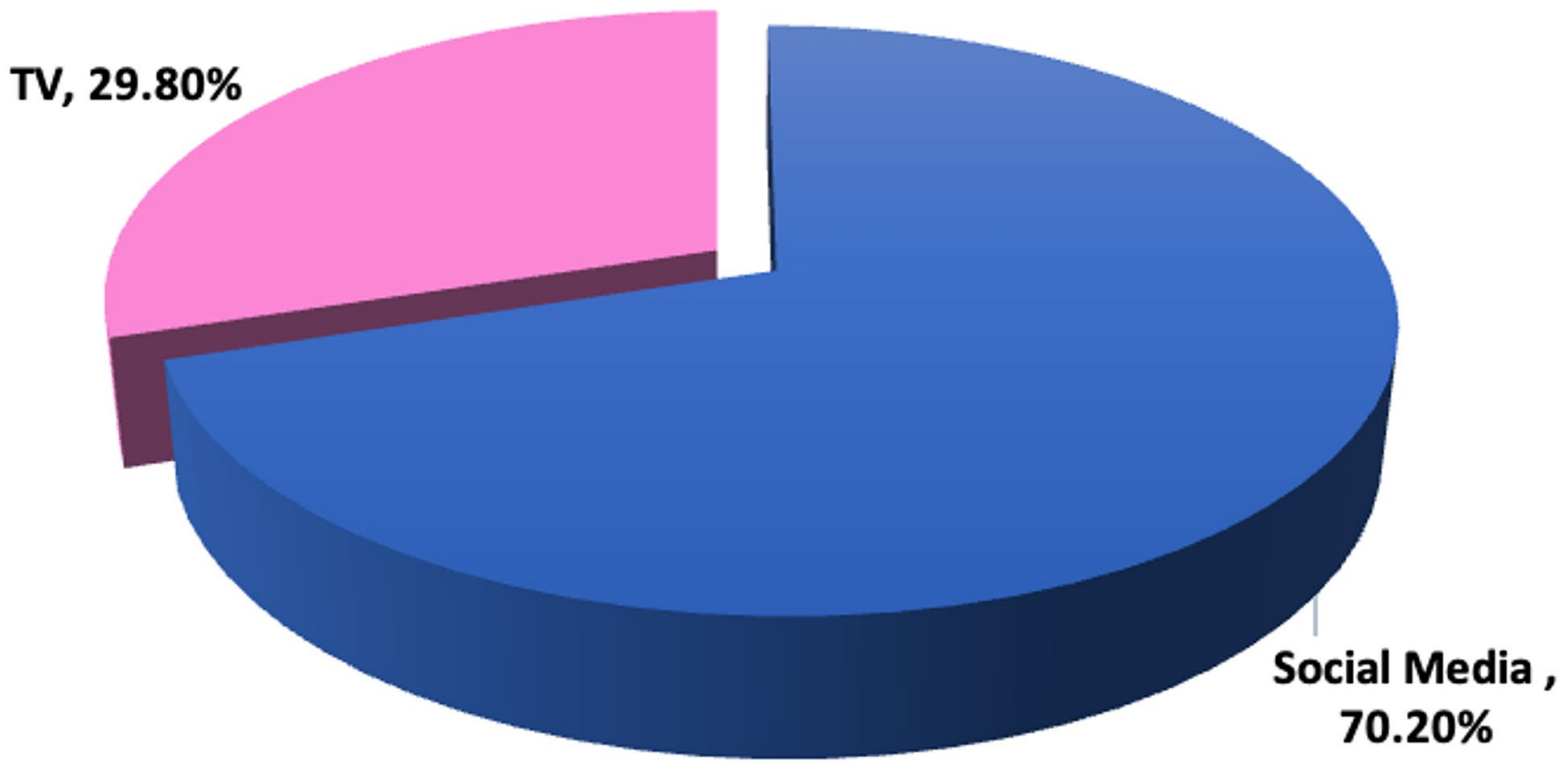
Media exposure channels of conflict scenes among studied school adolescents (*n* = 519)

Figure 2 shows that 70.2% of students viewed the events through various social media platforms (Instagram, Facebook, etc.), while 29.8% followed the events through Television programs.

**Fig. 3:**
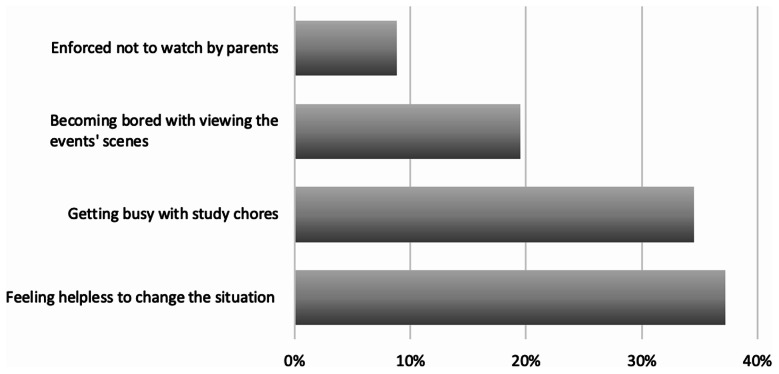
Factors made the studied school adolescents disengage with the conflict events (*n* = 519)

Figure 3 displays the reasons beyond cessation of following these events, where more than one-third felt helpless and powerless to change this precarious situation, and about another one-third got busy with their studying chores. While 19.5% of them got bored with following the news,and 8.8% were forced to stop watching by their parents.

Tables 2, 3 and 4 analyzed the relationship between psychological problems and sociodemographic characteristics. The univariate analysis detected statistically significant increased levels of stress, depression, and anxiety among females (40.3%, 74.8%, and 70.5%, respectively) than males and high school participants who were aged 15–18 years old, with prevalence rates of (35.8%, 68.1%, 60.3% respectively). Furthermore, adolescents from rural areas significantly experienced higher rates of mild and moderate stress levels (23.6% and 11.6%) than those of urban origin (18.5% and 4.4%). Unlikely, urban adolescents demonstrated higher rates of severe and extremely severe levels of anxiety (21.2%and 3.4%) than their rural counterparts (13.7% and 0.4%), with statistically significant differences.

**Table 2:** Relationship between sociodemographic characteristics and development of stress among the studied school adolescents (*n* = 519)

Sociodemographic	Stress level								X^2^	*p*-value
	Normal		Mild		Moderate		Total			
	*n*	%	*n*	%	*n*	%	*n*	%		
**Gender**										
Male	210	80.5%	39	14.9%	12	4.6%	261	100%		
Female	154	59.7%	72	27.9%	32	12.4%	258	100%	27.501	< 0.0001*
**Education Grade**										
Preparatory (12–14 y)	151	80.7%	26	13.9%	10	5.3%	187	100%		
Secondary (15–18 y)	213	64.2%	85	25.6%	34	10.2%	332	100%	15.729	0.0001*
**Residence**										
Urban	175	77.1%	42	18.5%	10	4.4%	227	100%		
Rural	189	64.7%	69	23.6%	34	11.6%	292	100%	12.248	0.002*
**Father’s occupation**										
Not working	49	80.3%	9	14.8%	3	4.9%	61	100%		
Working	315	68.8%	102	22.3%	41	9.0%	458	100%	3.479	0.179
**Mother’s occupation**										
Not working	204	66.2%	73	23.7%	31	10.1%	308	100%		
Working	160	75.8%	38	18.0%	13	6.2%	211	100%	5.792	0.055
**Education of parents**										
Illiterate	28	71.8%	8	20.5%	3	7.7%	39	100%		
Middle	148	74.0%	40	20.0%	12	6.0%	200	100%	3.798	0.429
High	188	67.1%	63	22.5%	29	10.4%	280	100%		

*X*^2^: Chi-square test, * Significant

**Table 3:** Relationship between sociodemographic data and development of anxiety among the studied school adolescents (*n* = 519)

Variable	Anxiety level												*p*-value
	Normal		Mild		Moderate		Severe		Extremely Severe		Total		
	*n*	%	*n*	%	*n*	%	*n*	%	*n*	%	*n*	%	
**Gender**													
Male	147	56.3%	22	8.4%	69	26.4%	20	7.7%	3	1.1%	261	100%	0.0001*
Female	76	29.5%	28	10.9%	73	28.3%	73	28.3%	8	3.1%	258	100%	
**Education Grade**													
Preparatory (12–14 y)	98	52.4%	18	9.6%	37	19.8%	33	17.6%	1	0.5%	187	100%	0.004*
Secondary (15–18 y)	125	39.7%	32	9.6%	105	31.6%	60	18.1%	10	3%	332	100%	
**Residence**													
Urban	111	38.00%	30	10.3%	79	27.1%	93	21.2%	10	3.4%	292	100%	0.009*
Rural	26	49.3%	5	8.8%	14	27.8%	7	13.7%	1	0.4%	227	100%	
**Mother occupation**													
Housewife	121	39.3%	33	10.7%	92	29.9%	56	18.2%	6	1.9%	308	100%	0.268
Working	102	48.3%	17	8.1%	50	23.7%	37	17.5%	5	2.4%	211	100%	
**Father occupation**													
Notworking	24	39.3%	7	11.5%	23	37.7%	6	9.8%	1	1.6%	61	100%	0.209
Employee	199	43.4%	43	9.4%	119	26.00%	87	19%	10	2.2%	458	100%	
**Parents education**													
illiterate	14	35.9%	5	12.8%	8	20.5%	12	30.8%	0	0.00%	39	100%	0.059
Middle	76	38%	20	10%	69	34.5%	31	15.5%	4	2%	200	100%	
High	133	47.5%	25	8.9%	65	23.2%	50	17.9%	7	2.1%	280	100%	

*X*^2^: Chi-square test, * Significant

**Table 4:** Relation between sociodemographic data and development of depression among the studied school adolescents (*n* = 519)

Socio-demographic	Depression level										X^2^	p-value
	Normal		Mild		Moderate		Severe		Total			
	n	%	n	%	n	%	n	%	n	%		
Gender												
Male	135	51.7%	72	27.6%	51	19.5%	3	1.1%	261	100%		
Female	56	25.2%	78	30.2%	109	42.2%	6	2.3%	258	100%	46.75	<0.000*
Education Grade												
Middle (12-14 y)	94	50.3%	51	27.3%	39	20.9%	3	1.6%	187	100%		
High (15-18 y)	106	31.9%	99	29.8%	121	36.4%	6	1.8%	332	100%	20.169	<0.000*
Residence												
Urban	107	36.6%	80	27.4%	98	33.6%	7	2.4%	292	100%		
Rural	93	41%	70	30.8%	62	27.3%	2	0.9%	227	100%	4.454	0.228
Father's occupation												
Not working	25	41%	19	31.1%	17	27.9%	0	0%	61	100%		
Working	175	38.2%	131	28.6%	143	31.2%	9	2%	458	100%	1.623	0.658
Mother's occupation												
Housewife	105	34.1%	101	32.8%	96	31.2%	6	1.9%	308	100%	8.080	0.047*
Working	95	45%	49	23.2%	64	30.3%	3	1.4%	211	100%		
Education of parents												
Illiterate	19	48.7%	8	20.5%	12	30.8%	0	0%	39	100%		
Middle	77	38.5%	62	31%	58	29%	3	1.5%	200	100%	3.838	0.699
High	104	37.1%	80	28.6%	90	32.1%	6	2.1%	280	100%		

*X*: Chi-square test, * Significant

Findings in tables 5, 6 and 7 unveiled statistically significant associations between watching conflict scenes with mild and moderate stress (22.1%,8.9%), moderate and severe depression (32.2%,1.8%), severe and highly severe anxiety (18.2,2.2%) compared to those who reported not watching any conflict related scenes (4.5%,0%), (0%,0%) and (4.5%, 0%) respectively. Moreover, adverse psychological problems were significantly associated with increased frequency and duration of watching conflict scenes since the proportion of the worst levels was higher among adolescents who used to watch the events for 5–7 days per week (44.7%, 69.7%, and 59.1%, respectively) and for more than 3 h per day (50%, 75%, and 69.4%, respectively) compared to others who reported lower frequency and duration.

**Table 5:** Relation between watching scenes of the Gaza war and the development of stress among the studied school adolescents (*n* = 519)

Variable	Stress level									
	Normal		Mild		Moderate		Total		X^2^	*p*-value
	*n*	%	*n*	%	*n*	%	*n*	%		
**Do you watch scenes of international conflict?**										
Yes	343	69.0%	110	22.1%	44	8.9%	497	100%	MCET	0.030*
No	21	95.5%	1	4.5%	0	0.0%	22	100%		
**Are you still watching these Conflicts?**										
Yes	269	66.3%	98	24.1%	39	9.6%	406	100%		
No	95	84.1%	13	11.5%	5	4.4%	113	100%	13.396	0.001*
**What are the average days you watch these scenes per week?**										
one day	43	81.1%	5	9.4%	5	9.4%	53	100%		
1–2 days	104	77.6%	25	18.7%	5	3.7%	134	100%	27.146	0.000*
3–4 days	123	69.1%	43	24.2%	12	6.7%	178	100%		
5–7 days	73	55.3%	37	28.0%	22	16.7%	132	100%		
**What are the average hours you watch these scenes per day?**										
< 1 h	109	71.7%	36	23.7%	7	4.6%	152	100%		
1–2 h	151	65.7%	54	23.5%	25	10.9%	230	100%	19.346	0.005*
2–3 h	65	82.3%	9	11.4%	5	6.3%	79	100%		
> 3 h	18	50.0%	11	30.6%	7	19.4%	36	100%		

* Significant *X*^2^: Chi-square test, MCET: Mont-Carlo Exact test

**Table 6:** Relation between watching scenes of the Gaza war and the development of depression among the studied adolescents (*n* = 519)

Variable	Depression level										X^2^	*p*-value
	Normal		Mild		Moderate		Severe		Total			
	*n*	%	*n*	%	*n*	%	*n*	%	*n*	%		
**Do you watch scenes of international conflict**												
Yes	179	36.0%	149	30.0%	160	32.2%	9	1.8%	497	100.0%	MCET	
No	21	95.5%	1	4.5%	0	0.0%	0	0.0%	22	100.0%	31.511	0.000*
**Are you still watching these Conflicts?**												
Yes	141	34.7%	123	30.3%	135	33.3%	7	1.7%	406	100.0%		
No	59	52.2%	27	23.9%	25	22.1%	2	1.8%	113	100.0%	11.817	0.008*
**What are the average days you watch these scenes per week?**												
One day	27	50.9%	12	22.6%	13	24.5%	1	1.9%	53	100.0%		
1–2 days	57	42.5%	43	32.1%	32	23.9%	2	1.5%	134	100.0%	19.950	0.020*
3–4 days	55	30.9%	52	29.2%	70	39.3%	1	0.6%	178	100.0%		
5–7 days	40	36.0%	42	30.0%	45	32.2%	5	3.8%	132	100.0%		
**What are the average hours you watch these scenes per day?**												
< 1 h	62	40.8%	42	27.6%	46	30.3%	2	1.3%	152	100.0%		
1–2 h	74	32.2%	65	28.3%	85	37.0%	6	2.6%	230	100.0%	15.733	0.069
2–3 h	34	43.0%	30	38.0%	5	19.0%	0	0.0%	79	100.0%		
> 3 h	9	25.0%	12	33.3%	14	38.9%	1	2.8%	36	100.0%		

*X*^2^: Chi-square test, MCET: Mont-Carlo Exact test, * Significance

**Table 7:** Relation between watching scenes of the Gaza war and the development of anxiety among the studied adolescents (*n* = 519)

Variable	Anxiety level												X^2^	*p*-value
	Normal		Mild		Moderate		Severe		Extremely Severe		Total			
	*n*	%	*n*	%	*n*	%	*n*	%	*n*	%	*n*	%		
**Do you watch scenes of international conflict?**														
Yes	204	41	50	10.1	140	28.2	92	18.5	11	2.2	497	100	MCET	0.004*
No	19	86.4	0	0	2	9.1	1	4.5	0	0	22	100		
**Are you still watching these Conflicts?**														
Yes	160	39.4	39	9.6	119	29.3	79	19.5	9	2.2	406	100		
No	63	55.8	11	9.7	23	20.4	14	12.4	2	1.8	113	100	10.637	0.03*
**What are the average days you watch these scenes per week?**														
One day	26	49.1	5	9.4	14	26.4	7	13.2	1	1.9	53	100		
1–2 days	53	39.6	17	12.7	37	27.6	26	19.4	1	0.7	134	100	6.075	0.919
3–4 days	71	39.9	14	7.9	53	29.8	35	19.7	5	2.8	178	100		
5–7 days	54	40.9	14	10.6	36	27.3	24	18.2	4	3	132	100		
**What are the average hours you watch these scenes per day?**														
< 1 h	71	46.7	12	7.9	37	24.3	30	19.7	2	1.3	152	100		
1–2 h	84	36.5	24	10.4	73	31.7	41	17.8	8	3.5	230	100	12.688	0.384
2–3 h	38	48.1	9	11.4	19	24.1	13	16.5	0	0	79	100		
> 3 h	11	30.6	5	13.9	11	30.6	8	22.2	1	2.8	36	100		

*X*^2^: Chi-square test, MCET: Mont-Carlo Exact test, * Significance

## Discussion

Childhood and adolescence represent an essential stage in developing mental health. More than half of mental health issues begin in these age groups, and many of these issues continue throughout adult life [32]. The psychological impact of watching war news, especially Middle East conflicts, has evolved between the past and the present. In the past, the traditional media and news were presented in a more controlled and organized manner, as not every home could watch what happened worldwide. Today, with increased access to online and social media news sources, individuals may feel a greater volume and diversity of information and gruesome images of frightening events [33, 34].

The present study aimed to assess the adverse effects of watching the ongoing war scenes of the Palestine-Israeli conflict in the Gaza Strip on adolescent’s mental health. The findings of the DASS-21 scales revealed that 29.9%, 61.5%, and 57% of respondents had variant degrees of stress, depression, and anxiety, respectively.

The current study found that the overall prevalence rates of stress, depression, and anxiety in our study were high, comparable to other studies conducted in different countries assessing the effect of the Russo-Ukrainian war on the same age group; in Poland (Stress 17.4%, Depression 29%, Anxiety 36.5%) and Taiwan (Stress 3.7%, Depression 11.2%, Anxiety 14.9%) [35].

There is evidence that neighbors who share the same language, beliefs, and religion as the inflicted people might have exacerbated reactive responses and become more sympathetic toward them [35, 36]. This might be the explanation for the observed high levels of psychological issues among our participants. Also, the geographical location can’t be ignored since Egypt borders Gaza in the northeast.

Interestingly, female adolescents experienced a statistically significant higher level of stress, depression, and anxiety than males. These results align with other studies, which showed that females had higher DASS-21 and IES-R (The Impact of Event Scale-Revised) scores [34]. Also, a systematic review included studies conducted between 1994 and 2014 interpreted that females who were not involved in a war zone in 30 countries were associated with a higher prevalence of depression [37]. Similar literaturee elucidated that the higher levels of mental health problems might be due to younger pubertal age, unhealthy ways of coping, including contemplation, and issues with peers and parenting, which make them more prone to emotional distress [38].

Though urban life confers its dwellers’ health facilities, better education, work opportunities, entertainment, and economic developments, it is deleterious to mental health [39]. Consistently, adolescents from urban areas demonstrated statistically significantly increased levels of anxiety than their peers from rural areas. Most evidence pointed out the detrimental effect of urban living on mental wellbeing, which has heightened levels of inequality, vehemence, racial or ethnic marginalization, environmental pollution, and diminished green space [39–41].

The present study revealed that adolescents in high schools who were aged between 15 and 18 years old experienced increased rates of stress 35.8%, depression 68.1%, and anxiety 60.3% comparable to their younger peers whose rates of stress, depression, and anxiety were 19.3%, 49.7%, and 47.6%. Studies suggest that when children get older, they might not seek social support, although they showed that social support has a mediating role in reducing mental health problems [42].

Social media have become an integral part of adolescents’ lives, with reports showing that 97% of adolescents are active on social media today [43]. Expectedly, most of the studied adolescents (70%) reported following the Gaza scenes across diverse social media platforms using their smartphones or devices, and mostly no parental guide for the watched frightening content. Millions of children and adolescents in the region are indirectly exposed to war, armed conflict, and terrorism through the media, inducing panic, fear, mass anxiety, constant hazard, and learned powerlessness [44].

The advancing, variant, accessible media channels, notorious for their amenability, render adolescents become insatiable users of media covering wars, armed confrontations, and terror attacks [45, 46].

Studies showed that the increased frequency, longer hours or intensity, and methods of media exposure determine the impact of watching conflict scenes on adolescents’ mental health, where highly gruesome images have exacerbated distress symptoms [47]. Consistently, our findings showed a significant prevalence of mental disorder symptoms among participants who reported watching the events for 5–7 days per week and more than 3 h per day compared to those who watched for less frequency and shorter duration. Additionally, previous literature demonstrated that actual violence has a more significant impact on children and adolescents than fictional ones, as the scenes could profoundly influence adolescents watched through different media channels portraying armed confrontations and incidents of terrorism [48]. Similarly, a study of the Polish population in the context of the war in Ukraine, covering about 72 participants, showed increased symptoms of depression and anxiety (64.7%,65%) among participants spending longer time watching the events. The authors explained fear for their future, the possible inclusion of their country in such war, and living in the same hostility and suffering [49].

Likely, another study finding about the National Survey of Stress Reactions after the September 11 terroristic attack on the United States showed that 35% of children had one or more stress symptoms, and 44% of adults reported one or more substantial symptoms of stress and were significantly associated with increased exposure time [50].

Our findings revealed that 70.2% of adolescents watching the scenes of the conflict were following the events through the different platforms on the Internet, while 29.8% were following the events on TV. Since the Internet provides quick access to real-time updates, it was no surprise that many students relied on it as their primary news source. Therefore, meticulous and supervised use of displayed materials on media could mitigate the risks of psychological distress among children and adolescents.

This observation is supported by a related study that detected that almost all of its participants (94.1%) reported looking at news online regularly. In comparison, 61% of participants watched news on TV but not frequently. Also, it referred to students’ tendency to get the news from the Iinternet as it allows them to check news between classes, while walking, and even during classes. Ffollowing multiple online news sources on social media such as CNN, The New York Times, USA Today, and the Huffington Post, with the convenience of their smartphones, they can look at their phones for news at any time [51].

About 21.8% of participants reported that they abruptly followed any scenes related to the Gaza conflict for multiple reasons. It was noted that the DASS-21 scale results were better among this group. The stress level doubled in adolescents still watching conflict scenes regularly (33.7%), comparable to 15.9% in adolescents who stopped watching the events (*p*-value = 0.001). Likely were depression and anxiety rates: 47.8% and 44.2% among students who stopped watching the events, while higher rates were observed among their counterparts who were still following the events regularly (65.3% and 60.6%, respectively).

Correspondingly, Slone et al.2013 concluded that intervention enhancement of self-mastery and refraining from direct exposure to war scenes were beneficial to decline the incidence of emotional distress symptoms among adolescents, compared to the control group who reported higher rates of psychological indices [52].

However, according to the theory of emotional desensitization, with repeated exposure to violence, people become “numb” to the violence, i.e., they experience less anxiety with each new exposure. However, this hypothesis might not apply to adolescence phases of life [53].

The present study investigated reasons explaining why adolescents stopped viewing conflict events; the results showed that more than one-third (37.2%) of participants stopped watching the events of conflict because they got busy with their studying chores, about one-third (34.5%) felt helpless to change the situation, 19.5% of them got bored with following the news and 8.8% were prohibited by their parents from watching these events.

Likely, a related study showed that people might take a break from the bad news because they might feel disempowered, helpless, apathetic, and even insensitive [52]. Another study showed that they might feel guilty as it suggested that individuals who follow situations in which people have died may develop “surveillance guilt” [54].

## Conclusion

The study unveiled that about one-third of adolescents were stressed, two-thirds were depressed, and about half were anxious. The findings also revealed a statistically significant association between watching scenes of war and the incidence of such mental health issues. In addition, the worst degrees of mental illness were significantly related to the intensity and duration of witnessing scenes through various media channels.

## Recommendation

This study provided a rationale for a comprehensive supportive approach addressing adolescents’ mental health needs, particularly in armed conflict zones. Controlling media exposure is a public health standing point since children and adolescents are highly vulnerable groups and the sole tax payers for any armed conflict.

### Strengths and limitations

According to the authors’ limited knowledge, this is the first study in the region measuring the effects of the Gaza conflict on the mental health of adolescents. Besides, this study targeted a substantial age group profoundly impacted by armed conflict scenes, which burdened their developmental complexity.

This study had several limitations. First, this cross-sectional study measured the immediate psychological outcomes though the Gaza-Israel war hasn’t been abrupted. Thus, further longitudinal study is required to monitor the long-term psychiatric and psychological sequelae and their association with post-war socio-economic status. Second, the sample size was relatively small (*n* = 519) due to limited resources. Finally, this study did not explore other psychiatric morbidities, including suicidal ideation or attempts, substance use, disturbed eating, and anti-social behavior. Further studies are required to assess the mental health of different age groups during the ongoing Gaza-Israel war and to assess mental well-being challenges.

## Data Availability

The datasets used and/or analysed during the current study are available from the corresponding author on reasonable request.

## Abbreviations

DASS: Depression Anxiety Stress Scale
WHO: World Health Organization
PTSD: Posttraumatic stress disorders
